# Evaluation of a secondary care multidisciplinary clinic for adults with early‐onset type 2 diabetes at high risk in Leicester, Leicestershire and Rutland

**DOI:** 10.1111/dme.70347

**Published:** 2026-04-30

**Authors:** Jonathan Goldney, Malak Hamza, Priscilla Sarkar, Harriet Morgan, Nicole Green, James Ridgeway, Victoria Alabraba, Laura Willcocks, Krupali Chandracant, Kashmala Javed, Rhys O'Callaghan, Finley Turner, Samuel Seidu, Claire L. Meek, Melanie J. Davies

**Affiliations:** ^1^ Diabetes Research Centre, Leicester General Hospital, College of Life Sciences University of Leicester Leicester UK; ^2^ NIHR Leicester Biomedical Research Centre University of Leicester & University Hospitals of Leicester NHS Trust Leicester UK; ^3^ Leicester Diabetes Centre, Leicester General Hospital University Hospitals of Leicester NHS Trust Leicester UK

**Keywords:** case finding, health services evaluation, multidisciplinary care, type 2 diabetes mellitus, young adult

## Abstract

**Background:**

Clinical services for adults with early‐onset type 2 diabetes (EOT2D) are in urgent need of improvement.

**Methods:**

We evaluated a multi‐disciplinary clinic for individuals with EOT2D at high risk (HbA1c >9%/triglyceride >20 mmol/L), providing 2–3 flexibly arranged consultations per individual between March 2024 and September 2025. Nurse‐led case‐finding in primary care supported identification and targeted referral of high‐risk individuals.

On 3 November 2025, electronic medical records were used to evaluate change in cardiometabolic outcomes at 6‐ and 12‐months from baseline and evaluate service activity, including proportions with recorded health information and prescriptions. The paired *t*‐test (continuous) and McNemar's test (proportions) were used to assess change in outcomes. Patient feedback was obtained using questionnaires.

**Results:**

In total, 159 individuals received an appointment (45% women; median age 34 years). At both 6‐ and 12‐months post‐baseline, HbA1c (at 12‐months: −2.53% [95% CI: −3.13, −1.93]/−28 mmol/mol [−34, 21]), systolic (−5.89 mmHg [−9.88, −1.91]) and diastolic (−2.72 mmHg [−5.34, −0.10]) blood pressure, body mass index (BMI; −1.49 kg/m^2^ [−2.31, −0.67]) and weight (−3.52 kg [−6.32, −0.72]) were significantly lower. Triglycerides were lower at 12‐months only (−1.12 mmol/L [−1.89, −0.34]). The proportion with BMI or smoking status recorded, and with a glucagon‐like peptide‐1 receptor‐based treatment, was higher at 12‐months than at baseline, whereas creatinine and retinal screening measurements were less frequent. A limited amount of patient feedback was positive.

**Discussion:**

Case‐finding and invitation to flexibly run multidisciplinary clinics were associated with improvements in cardiometabolic outcomes and positive patient feedback. Similar services are immediately required given the rising prevalence of EOT2D and associated complications.

**What's new?**

- Adults with early‐onset type 2 diabetes (EOT2D) experience high complication risk and poor attainment of treatment targets, yet existing services are often poorly aligned to their clinical and social needs.
- We evaluated a pragmatically designed, multi‐disciplinary clinic using case‐finding, flexible delivery and person‐centred care to improve engagement and outcomes in individuals with high‐risk EOT2D. The service was associated with meaningful improvements in glycaemic control, body weight and blood pressure, alongside a limited number of positive patient feedback.
- This evaluation provides implementable, real‐world evidence to inform scalable redesign of EOT2D services within routine healthcare systems.

## 1 INTRODUCTION

The prevalence of early‐onset type 2 diabetes (EOT2D), defined as diagnosis before 40 years of age, is increasing in the United Kingdom and globally.^1^, ^2^ EOT2D is associated with an extremely high‐risk phenotype.^3^ Individuals with EOT2D have higher glucose levels at diagnosis and throughout the disease course compared with those with later‐onset disease, resulting in more than threefold higher cumulative glycaemic exposure by age 75 years.^4^ Consequently, EOT2D is associated with a high risk of microvascular and macrovascular complications, premature mortality and multiple long‐term conditions.^5^, ^6^, ^7^, ^8^ Beyond traditional diabetes complications, individuals with EOT2D have high rates of psychiatric comorbidity, adverse psychosocial well‐being, diabetes‐related stigma and pregnancy‐related complications.^9^, ^10^, ^11^, ^12^, ^13^

There is a clear need to improve the clinical care of adults with EOT2D. However, diabetes services remain largely structured around later‐onset disease, with fragmented pathways and limited flexibility to meet the complex clinical and psychosocial needs of this group. In England, the National Diabetes Audit demonstrated that individuals with EOT2D are substantially less likely to achieve recommended HbA1c targets or complete nationally determined care processes compared with older adults^14^; a pattern also observed internationally.^15^, ^16^ Individuals with EOT2D are less likely to take prescribed medications or attend appointments.^17^, ^18^ Engaging with healthcare services which are often inflexible can be challenging for adults with EOT2D. There are multiple potential reasons for this including employment pressures in early careers; lack of flexibility in less‐skilled, lower‐income or unstable employment; being in full‐time education; having young families; adverse mental health; or higher rates of stigma. New models of care tailored to this population are therefore required.

Consequently, the T2Day programme was launched by NHS England in 2023, a national programme to provide financial support to Integrated Care Boards (ICBs; the regional commissioners for health services in England) to improve care for adults with EOT2D.^19^ The allocated funds corresponded to approximately £52 per individual with EOT2D per year over two years and could be flexibly spent where necessary regionally. In Leicester, Leicestershire and Rutland, we used these funds to design a regional service for adults with EOT2D, described previously.^20^ A core component included clinics for individuals deemed to be at high clinical risk. In this manuscript, we comprehensively evaluated these high‐risk clinics utilising electronic medical records (EMRs) to review clinical outcomes and activity, and obtain user feedback.

## 2 METHODS

### 2.1 Overview of the high‐risk clinic

In Leicester, Leicestershire and Rutland, between March 2024 and September 2025, specialist clinics for adults with EOT2D deemed at high‐risk were run in secondary care. Individuals were eligible for the clinic if they had EOT2D, an age at time of referral <40 years and an HbA1c ≥10%/86 mmol/mol or a triglyceride level ≥20 mmol/mol. The HbA1c threshold was lowered around halfway through the service in December 2024 to 9%/75 mmol/mol due to surplus capacity. Communications were sent to all GP practices to refer individuals via a simple refined electronic referral system which required no free‐text. As described previously,^20^ the broader service involved diabetes specialist nurses (DSNs) visiting practices with a high number of individuals with EOT2D and assisting with the identification of eligible individuals and inviting them to the service using pre‐built EMR searches.

The clinics incorporated a multi‐disciplinary team approach consisting of diabetologists, DSNs and dieticians. Underpinning the clinics was a person‐centred approach recognising the unique life circumstances of adults with EOT2D. This included allowing individuals to choose the timing of appointments, including out‐of‐hours when required; contacting individuals via their preferred contact method; being understanding of non‐attendance, with no policy discharging individuals for non‐attendance; having a single point of contact for clinics; providing reminders of appointments; holding telephone, virtual and face‐to‐face formats based on individual preference; and undertaking consultations in an individual's preferred spoken language via translation services or facilitated through the breadth of languages spoken by the clinical team.

The clinics were initially intended to be ‘one‐stop’; however, in practice, this consisted of two or three consultations per individual to provide a greater level of support than a single appointment. After the final consultation, the individual would be either (a) discharged back to primary care with advice for further management; (b) referred for further support in secondary care clinics; or (c) enrolled in a suitable clinical trial with ongoing support from the clinical trial team.

The remit of the clinic was wide and included management of hyperglycaemia and other cardiovascular risk factors such as weight, lipids and blood pressure; screening for complications; women's health, with referral to the preconception clinic similarly funded by the T2Day programme where appropriate; and psychological well‐being, with referral via pre‐existing pathways for psychological interventions. To support consultations, individuals attending their initial consultation were asked to complete optional questionnaires, including the Patient Health Questionnaire‐9 (PHQ9), the EuroQol‐5D‐5L (EQ‐5D‐5L) and the Problem Areas in Diabetes (PAID).

For evaluation purposes, the cohort included any individual who had a letter written within the service; this included a few individuals with booked appointments but who did not attend. This approach reflects real‐world service delivery, referral effectiveness and engagement challenges inherent to EOT2D care.

As this work was undertaken as part of routine service evaluation, written consent and ethical approval were not required.

### 2.2 Outcomes determined by EMRs

On the 3 November 2025, EMRs were used to evaluate the following outcomes: HbA1c, low‐density lipoprotein (LDL) cholesterol, high‐density lipoprotein (HDL) cholesterol, serum or plasma triglyceride, serum creatinine, urinary albumin: creatinine ratio (UACR), systolic/diastolic blood pressure, body weight and body mass index (BMI). These outcomes were identified both at baseline, 6 months and 12 months. A baseline measurement was defined as the most recent recorded value within 1 year before or 1 week after the first clinic letter (assumed to be the date of the first consultation). A 6‐month measurement was defined as the recorded value closest to 6 months but within 3–9 months after the first clinic letter, with 12 months defined as the closest within 9–15 months.

EMRs were also used to evaluate service activity. This included the number of individuals meeting national care processes for the collection of important health data within the prior 12 months,^21^ both at baseline and 12 months; and also the number of people with a relevant prescription for lipid lowering therapy, antihypertensives, folic acid (in women, reflecting an aspect of preconception care) or each class of glucose‐lowering therapy in the prior 4 months.

We also used EMRs to identify individuals with an alternative type of diabetes recorded after the first appointment in the clinic, namely type 1 diabetes, maturity‐onset diabetes of the young (MODY) or latent autoimmune diabetes in adults (LADA). Similarly, as large reductions in HbA1c are associated with incident microvascular disease, we compared the proportion of individuals with microvascular complications at baseline and 12 months, including EMR recorded retinopathy, neuropathy, chronic kidney disease, or measured UACR >3 mmol/mol (albuminuria).

EMRs were also used to identify demographic characteristics to support reporting on the representation of various groups within our service. The Index of Multiple Deprivation (IMD) 2019 was calculated for each individual using their postal code and was categorised into quintiles. Ethnicity codes were obtained and grouped into Black, South Asian, White, Other and Unknown for reporting purposes (codes presented in Table S1). Similarly, EMRs were used to report past medical history and most recently recorded smoking status prior to baseline. Type 2 diabetes duration was calculated as the difference between the first recorded diabetes code and the date of the first clinic letter.

### 2.3 Patient feedback

On 18 June 2025, individuals with EOT2D that had attended at least one consultation within the service were sent a text message inviting them to complete an anonymous online survey about their experience via a secure Microsoft Form. This included four questions; the first three were graded on a Likert scale of 1 to 5, the fourth question included a free‐text box:

- How would you rate the overall quality of care you received within the service?
- Has the service helped you to manage your diabetes better?
- Did the way we arranged your appointment work well for you?
- Is there any way we could improve the service? Is there anything else you would like to say about the service?

### 2.4 Statistical analysis

We reported the total number of referrals received in the service as compared to the estimated number that were eligible at the outset, which was obtained from EMR searches undertaken prior to the start of the service previously described.^20^ We similarly reported the number of practices visited by DSNs, individuals contacted and individuals referred through this activity.

We summarised baseline characteristics including demographics, past medical history, biochemistry and current medications. Continuous measures were reported as median (interquartile range [IQR]), and categorical measures were reported as number (% of total).

We calculated the difference in cardiometabolic outcomes between baseline and 6 months and baseline and 12 months separately, for individuals with available data for both timepoints (paired data), using the paired *t*‐test to calculate *p*‐values and 95% confidence intervals (CI). Similarly, we reported the number (% of those with >12‐months follow‐up) of individuals meeting care processes, with HbA1c below certain thresholds (<6.5%/48 mmol/mol, <7.5%/58 mmol/mol, <8.5%/69 mmol/mol), taking each class of medication and with reported microvascular outcomes at baseline and 12‐months; McNemar's test was used to compare the proportions for each outcome across these time points to calculate *p*‐values. The proportion of individuals with national care processes recorded was reported alongside the latest published national benchmarks.^22^, ^23^ No imputation was performed for missing follow‐up data; analyses were restricted to available paired observations, consistent with the service evaluation design.

We further reported the responses of individuals to the patient feedback questions in crude terms.

For all analyses, *p* < 0.05 was the threshold for statistical significance. All analyses were undertaken in Stata v19.

## 3 RESULTS

### 3.1 Total referrals

Overall, 203 eligible individuals were referred to the high‐risk clinic (Figure 1). Referrals came from primary care directly (*n* = 162) and through DSN‐led case‐finding (*n* = 41). The DSN team visited 21/30 of the primary care practices with the highest number of individuals with EOT2D in the ICB. During these visits, the DSN team contacted 129 individuals directly, leading to 41 referrals received (20% of total). Of the 203 individuals referred, 44 were either not able to be contacted or withdrew interest and so never received an appointment, with 159 individuals receiving an appointment for an initial consultation and included in analyses. Out of 159 individuals, approximately 150 attended at least one appointment and 9 individuals had an appointment(s) scheduled but never attended.

**FIGURE 1:**
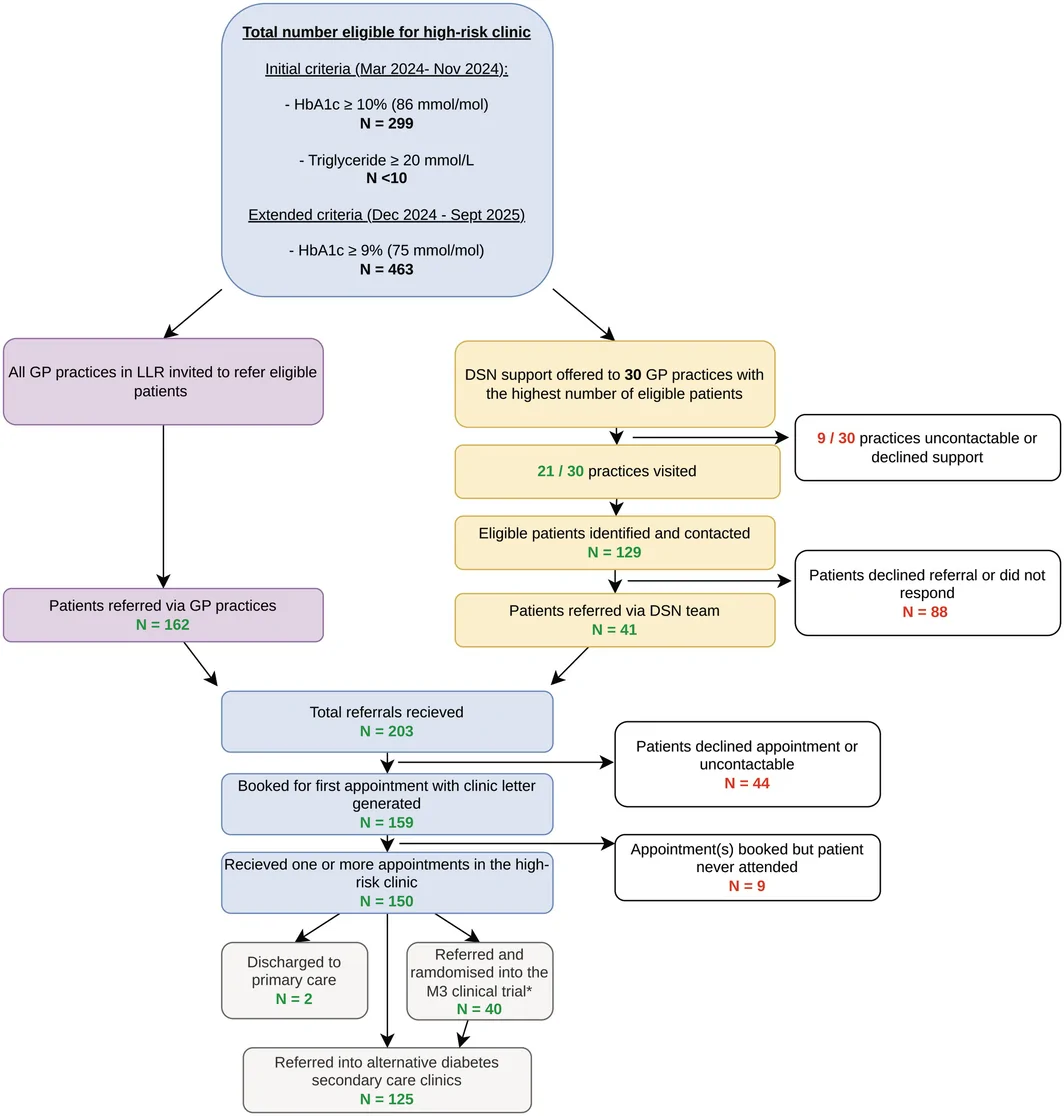
Flow of individuals seen in the high‐risk clinic. All numbers represent approximations as data was pulled retrospectively from electronic medical records (EMR). The total number eligible was obtained from regional EMR searches on the 15th November 2023, described previously.^20^ *The M3 clinical trial is an ongoing randomised controlled trial in which individuals with EOT2D can receive ongoing secondary care support amongst a range of novel treatment options within an alternative model of care (more details at: https://www.m3study.org.uk/). Individuals in the M3 trial are randomised 1:1 to usual care or intervention. Discharge or follow plans were made for those randomised to control. DSN: diabetes specialist nurse; GP practice: primary care (general practitioner) practice; LLR: Leicester, Leicestershire and Rutland.

### 3.2 Baseline characteristics

Table 1 shows the baseline characteristics of our cohort. Overall, there were fewer women than men, 70 (45%) versus 86 (55%) respectively, with a median (IQR) age of 34 years (30, 39). Most individuals were from the top two most deprived quintiles, 28% from Quintile 1 and 37% from Quintile 2. Individuals were from a range of ethnic groups, with the largest proportion being from South Asian (44%), then White (29%), Other (12%) and Black (6.4%) ethnic groups. The median (IQR) duration of type 2 diabetes was relatively short at 2.4 years (0.2, 7.0). Baseline cardiometabolic risk appeared to be high with a median (IQR) HbA1c of 10.7%/93 mmol/mol (9.4–12.0%/79–108 mmol/mol), BMI of 33 kg/m^2^ (29, 39) and triglycerides of 2.1 mmol/L (1.4, 3.6). The most prevalent baseline comorbidities included anxiety (24%), depression (24%), hypertension (15%) and dyslipidaemia (12%). The most prescribed glucose‐lowering medications were metformin (72%), insulin (30%) and sulphonyl ureas (21%); with 22% on antihypertensives and 28% on lipid‐lowering therapy.

**TABLE 1:** Baseline characteristics.

Characteristic	Value	No. of individuals with available data (% total)
Age (years)	34 (30, 39)	156 (100)
Sex		
Women	70 (45)	156 (100)
Men	86 (55)	
Deprivation		
Most deprived—Quintile 1	43 (28)	156 (100)
Quintile 2	57 (37)	
Quintile 3	30 (19)	
Quintile 4	14 (9)	
Least deprived—Quintile 5	12 (7.7)	
Ethnicity		
Black	10 (6.4)	—
South Asian	68 (44)	
White	46 (29)	
Other	18 (12)	
Unknown (including missing)	14 (9)	
Type 2 diabetes duration (years)	2.4 (0.2, 7.0)	152 (97)
Smoking status		
Smoker	<5	Suppressed
Non‐smoker	<5	
Ex‐smoker	6 (21)	
Never smoked tobacco	19 (66)	
Patient‐reported outcomes		
EQ‐5D‐5L (total score^a^)	8 (5, 11)	40 (26)
EQ‐5D‐5L (visual analogue score)	55 (36, 88)	40 (26)
PHQ9 (score)	12 (6, 20)	45 (29)
PAID (score)	12 (9, 17)	43 (28)
Anthropometrics		
BMI (kg/m^2^)	33 (29, 39)	124 (79)
Weight (kg)	94 (80, 111)	122 (78)
Blood pressure		
Systolic (mmHg)	127 (119, 136)	123 (79)
Diastolic (mmHg)	83 (78, 90)	127 (81)
Biochemistry		
HbA1c (%)	10.7 (9.4, 12.0)	145 (93)
HbA1c (mmol/mol)	93 (79, 108)	
LDL (mmol/L)	2.5 (1.9, 3.1)	122 (78)
HDL (mmol/L)	1.0 (0.9, 1.2)	145 (93)
TG (mmol/L)	2.1 (1.4, 3.8)	138 (88)
Creatinine (μmol/L)	71 (61, 82)	118 (76)
UACR (mg/mmol)	2.0 (0.8, 8.1)	51 (33)
UACR >3 mg/mmol	22 (43)	
Past medical history		
Amputation	<5	
Anxiety	37 (24)	
Depression	37 (24)	
Previous diabetic ketoacidosis	<5	
Dyslipidaemia	19 (12)	
Erectile dysfunction (% of men)	<5	
Hypertension	23 (15)	
Ischaemic heart disease	<5	
Learning disability	<5	
Peripheral neuropathy	<5	
Psychosis	<5	
Retinopathy	6 (3.8)	
Ulcer	<5	
Chronic kidney disease	<5	
Hospital attendance in last year	<5	
Medications		
DPP4 inhibitor	8 (5.1)	
Sulphonyl urea	32 (21)	
GLP‐1 RA	27 (17)	
Insulin	47 (30)	
Metformin	113 (72)	
SGLT2i	28 (18)	
Tirzepatide	7 (4.5)	
Antihypertensives	35 (22)	
Lipid‐lowering therapy	44 (28)	
Folic acid (% of women)	9 (13)	

*Note*: In accordance with NHS information governance standards, low counts (<5) were suppressed to maintain anonymity.Continuous measures are shown as median (interquartile range) and categorical numbers as counts (% of total, unless otherwise stated).

Abbreviations: BMI, body mass index; DPP4, dipeptidyl peptidase‐4; EQ‐5D‐5L, EuroQol 5‐Dimension 5‐Level; GLP‐1 RA, glucagon‐like peptide‐1 receptor agonist; HDL, high‐density lipoprotein cholesterol; LDL, low‐density lipoprotein cholesterol; PAID, Problem Areas in Diabetes scale; PHQ‐9, Patient Health Questionnaire‐9; SBP, systolic blood pressure; SGLT2i, sodium–glucose cotransporter‐2 inhibitor; TG, triglycerides; UACR, urine albumin‐to‐creatinine ratio.

^a^ Reporting the EQ‐5D‐5L by domain was not possible within electronic medical records.

### 3.3 Change in cardiometabolic outcomes

Table 2 shows changes in the cardiometabolic outcomes at 6‐ and 12‐months from baseline for individuals with data at both time points (median days between first visit and outcome measurement shown in Table S2). For individuals with available data, at 6‐ and 12‐months, HbA1c was significantly lower than at baseline, −2.53% (−3.13, −1.93)/−28 mmol/mol [−34, −21] lower at 12‐months. Similarly, the proportion of individuals achieving HbA1c <7.5%/58 mmol/mol improved from 6.9% at baseline to 35.4% at 6‐months and 38.0% at 12‐months (*p*‐difference <0.001 at both timepoints) with similar improvements in the proportion achieving HbA1c <6.5%/48 mmol/mol and <8.5%/69 mmol/mol (Table S3).

**TABLE 2:** Change in cardiometabolic outcomes from baseline.

Outcome	6 months					12 months				
	Baseline value	12‐month value	Change from baseline (95% CI)	*p*‐value	Pairs (% of total)	Baseline value	12‐month value	Change from baseline (95% CI)	*p*‐value	Pairs (% of total)
HbA1c (%)	10.38 (2.01)	8.64 (2.28)	**−1.74 (−2.17, −1.32)**	**<0.001**	109 (69.9%)	10.74 (1.88)	8.21 (2.18)	**−2.53 (−3.13, −1.93)**	**<0.001**	68 (43.6%)
HbA1c (mmol/mol)	90 (22)	71 (25)	**−19 (−23, −14)**	**<0.001**	109 (69.9%)	94 (21)	66 (24)	**−28 (−34, −21)**	**<0.001**	68 (43.6%)
LDL (mmol/L)	2.61 (0.98)	2.40 (0.83)	−0.21 (−0.42, 0.00)	0.052	73 (46.8%)	2.59 (0.84)	2.62 (0.86)	0.02 (−0.26, 0.30)	0.874	36 (23.1%)
HDL (mmol/L)	1.05 (0.29)	1.04 (0.21)	−0.01 (−0.07, 0.04)	0.673	52 (33.3%)	1.06 (0.32)	1.06 (0.22)	−0.00 (−0.11, 0.10)	0.955	24 (15.4%)
TG (mmol/L)	3.23 (3.33)	2.57 (2.68)	−0.66 (−1.33, 0.00)	0.052	86 (55.1%)	3.76 (3.45)	2.64 (2.86)	**−1.12 (−1.89, −0.34)**	**0.006**	43 (27.6%)
Creatinine (μmol/L)	69.93 (16.04)	72.36 (13.08)	2.43 (−2.55, 7.42)	0.322	22 (14.1%)	. (.)	. (.)	‐		
UACR (mg/mmol)	14.35 (20.64)	5.52 (7.66)	−8.83 (−18.97, 1.32)	0.083	14 (9.0%)	6.17 (6.78)	4.80 (6.29)	−1.37 (−5.93, 3.19)	0.490	7 (4.5%)
SBP (mmHg)	127.80 (13.92)	123.52 (11.99)	**−4.29 (−7.37, −1.21)**	**0.007**	93 (59.6%)	129.94 (14.93)	124.05 (14.40)	**−5.89 (−9.88, −1.91)**	**0.004**	58 (37.2%)
DBP (mmHg)	83.95 (8.87)	81.64 (8.07)	**−2.32 (−4.50, −0.13)**	**0.038**	95 (60.9%)	84.59 (9.42)	81.88 (8.66)	**−2.72 (−5.34, −0.10)**	**0.042**	58 (37.2%)
BMI (kg/m^2^)	34.33 (8.32)	33.13 (7.48)	**−1.20 (−1.99, −0.40)**	**0.004**	93 (59.6%)	34.31 (7.80)	32.82 (7.66)	**−1.49 (−2.31, −0.67)**	**0.001**	56 (35.9%)
Weight (kg)	95.35 (27.72)	92.77 (25.69)	**−2.59 (−5.06, −0.11)**	**0.041**	94 (60.3%)	97.40 (28.58)	93.88 (25.45)	**−3.52 (−6.32, −0.72)**	**0.015**	59 (37.8%)
Weight (%)	100.00 (0.00)	98.57 (14.28)	−1.43 (−4.36, 1.49)	0.334	94 (60.3%)	100.00 (0.00)	97.88 (13.27)	−2.12 (−5.58, 1.33)	0.224	59 (37.8%)

*Note*: Data are summarised for individuals with data at both timepoints meaning there are small differences in baseline values for individuals with complete data at 6‐months and 12 months. Change in creatinine at 12 months could not be calculated due to <2 pairs. Values represent mean (SD). Statistically significant (*p* < 0.05) results are highlighted in bold.

Abbreviations: BMI, body mass index; HDL, high‐density lipoprotein cholesterol; LDL, low‐density lipoprotein cholesterol; SBP, systolic blood pressure; TG, triglycerides; UACR, urine albumin‐to‐creatinine ratio.

In addition to HbA1c reduction, at 12‐months, there were also reductions in systolic blood pressure, −5.89 mmHg (−9.88, −1.91); diastolic blood pressure, −2.72 mmHg (−5.34, −0.10); BMI, −1.49 kg/m^2^ (−2.31, −0.67); weight, −3.52 kg (−6.32, −0.72); and triglycerides, −1.12 (−1.89, −0.34). Systolic and diastolic blood pressure, BMI, and weight (kg) were also significantly lower at 6‐months. Other cardiometabolic outcomes were not significantly different; however, there was a non‐significant trend towards improvement in most outcomes at both timepoints.

### 3.4 Service activity measures

Table 3 shows changes in the number of individuals with complete care processes and the medications received by individuals at baseline and 12‐months. There were a total of 82 individuals (53% of total) with follow‐up duration >12 months (the duration between the first consultation and date of analysis). The proportion of individuals with recorded care processes was variable across individual care processes and time points, and most were lower than the latest national benchmark. Compared to baseline, at 12‐months there was a significantly higher number of individuals with BMI (80% vs. 91% respectively) and smoking status (9% vs. 46%) recorded and a significantly lower number with creatinine (89% vs. 48%) and an eye screen (49% vs. 26%) recorded. For the remaining outcomes, there were no significant differences in the proportions of individuals with recorded care processes at baseline vs. 12‐months.

**TABLE 3:** Service activity measures.

Outcome	Baseline	12‐months	*p*‐value for difference	National benchmark (%)^a^
Care processes measured in the last 12 months				
BMI	66 (80)	75 (91)	**0.04**	87
Cholesterol	69 (84)	71 (87)	0.83	87
Creatinine	73 (89)	39 (48)	**0.00**	91
SBP	70 (85)	75 (91)	0.30	92
HbA1c	76 (93)	73 (89)	0.55	92
UACR	29 (35)	37 (45)	0.27	69
Smoking	7 (8.5)	38 (46)	**0.00**	94
Foot screen	55 (67)	50 (61)	0.44	78
Eye screen	40 (49)	21 (26)	**0.01**	81
Current medication				
DPP4 inhibitor	<5	5 (6.1)	1.00	—
Sulphonyl urea	14 (17)	12 (15)	0.77	
GLP‐1 RA	14 (17)	26 (32)	**0.03**	
Insulin	32 (39)	32 (39)	1.00	
Metformin	60 (73)	54 (66)	0.29	
SGLT2i	13 (16)	13 (16)	1.00	
Tirzepatide	<5	16 (20)	**0.00**	
Antihypertensive	24 (29)	28 (34)	0.39	
Lipid‐lowering therapy	26 (32)	31 (38)	0.30	
Folic acid^b^	<5	<5	—	

*Note*: Baseline and 12‐month data are presented as counts (% of total number of patients with at least 1‐year of follow up; *n* = 82). In accordance with NHS information governance standards, low counts (<5) were suppressed to maintain anonymity, however *p*‐values for difference were still calculated. Care‐processes were defined as the recording of each outcome within the prior 12‐months. Based on guidelines for care in England, see: https://www.nice.org.uk/guidance/qs209/chapter/Quality‐statement‐6‐9‐key‐care‐processes. Current medication was defined as a prescription within the prior 4‐months within the relevant group of medications. Statistically significant results are highlighted in bold.

Abbreviations: BMI, body mass index; DPP4, dipeptidyl peptidase‐4; GLP‐1 RA, glucagon‐like peptide‐1 receptor agonist; SBP, systolic blood pressure; SGLT2i, sodium–glucose cotransporter‐2 inhibitor; UACR, urine albumin‐to‐creatinine ratio.

^a^ National benchmarks were obtained from the latest published NHS England data.^22^, ^23^

^b^ Out of the 82 individuals in the service for >12 months at time of analysis, there were only 34 women (48 men), <5 of these women had folic acid prescription at either time point.

After a 12‐month follow‐up, the number of individuals taking glucagon‐like peptide‐1 receptor agonists (GLP‐1 RAs) and tirzepatide had increased: from 17% to 32% for GLP‐1 RAs and <5 (<6%) to 20% for tirzepatide. For other classes of medications, while there were numerous individual‐level changes, for example, insulin was discontinued in 5 (6%) and initiated in another 5 (6%) of individuals, there were no significant differences in the aggregate proportions taking each class of medication across the two timepoints. For folic acid, <5 individuals were recorded at either baseline or 12‐month time points.

### 3.5 Additional outcomes of interest

In the 82 individuals with follow‐up duration >12 months, <5 individuals were reclassified as having type 1 diabetes, MODY or LADA. Similarly, for microvascular complications, <5 individuals developed retinopathy, peripheral neuropathy or had new chronic kidney disease coded, although as demonstrated in Table 2, screening for complications was variable. There were similar proportions with evidence of albuminuria (UACR >3 mg/mmol) at each time point: 22 individuals with a positive result out of 51 individuals with UACR measured (43.1%) at baseline, 12/30 (40.0%) at 6‐months and 8/18 (44.4%) at 12‐month (*p*‐difference >0.05 at both timepoints).

### 3.6 Patient feedback

In total, 25 individuals responded to our request for feedback. For the first three questions related to overall quality of care, management of diabetes and appointment arrangement, the majority of responses were positive (Figure 2), with 68%, 64% and 64% responding with 5/5, respectively. Similarly, 10 respondents provided free‐text responses to question 4, which were generally positive (Table S4).

**FIGURE 2:**
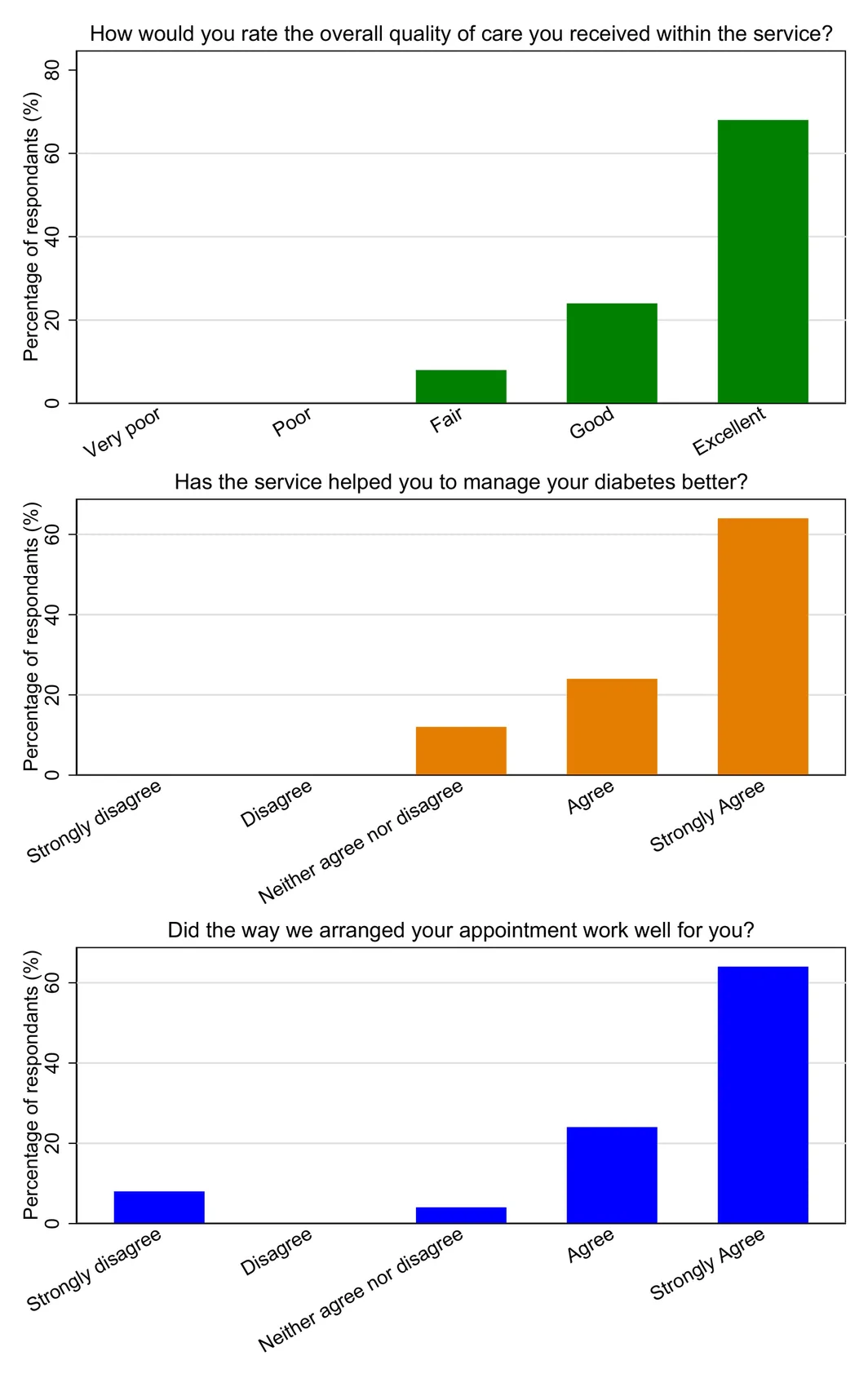
Patient feedback. Bar charts show questionnaire feedback from 25 patients who responded.

### 3.7 Further management

After receiving at least one appointment in the high‐risk clinic (Figure 1), 2 patients were discharged with advice to primary care, 40 were referred and randomised into relevant clinical trials (some of which still required follow up plans if receiving control arms of an intervention) and 125 were referred into other appropriate secondary care clinics.

## 4 DISCUSSION

Overall, we demonstrated that individuals with EOT2D at high risk of complications generally had clinical improvements while within our service. This included improvements in HbA1c (−2.53%/−28 mmol/mol), BMI (−1.50 kg/m^2^), weight (−3.52 kg), triglycerides (−1.12 mmol/l) and systolic (−4.22 mmHg) and diastolic (−2.72 mmHg) blood pressure at 12‐months from baseline in individuals with available data. A limited amount of patient feedback supported positive user satisfaction within our service.

Our findings are encouraging that the disease trajectory of individuals with EOT2D at high risk of complications may be modifiable. While glucose levels are often lower soon after diagnosis of EOT2D, likely related to initiation of glucose‐lowering therapies and lifestyle changes, real‐world data across several countries demonstrate that individuals with EOT2D have consistently elevated glucose as compared to individuals with later‐onset throughout the disease course.^4^, ^24^, ^25^

### 4.1 Strengths and limitations

This evaluation is strengthened using EMRs, with up to 1‐year follow‐up and obtaining patient feedback. The evaluation is also highly novel, timely and relevant due to the increasing burden of EOT2D and lack of previously described service‐level interventions.

However, this evaluation was not undertaken as research and so there was no control arm, meaning that causality cannot be inferred. Furthermore, our reliance on routinely collected data meant that there was considerable missing data, which was largely contributed to by only 82 (53%) individuals having their first consultation at least 1 year prior to the date of evaluation. This is a balance between faster evaluation and sharing of findings to potentially allow adoption and improvement of care elsewhere, or delaying evaluation to improve completeness of data. The small number of individuals may have limited statistical power to detect differences in outcomes across different timepoints. Our person‐centred approach may also have caused missing data as clinicians were mindful that obtaining regular biochemical measures can add burden to individuals with EOT2D that already have busy lives, early careers, young families, low income and a high burden of mental health. These factors may be even more relevant to individuals with EOT2D and high HbA1c. The low completeness of data and small sample size is likely to have contributed to the low counts reported developing microvascular complications and having diabetes reclassified. While we recognise that screening for complications is important, we prioritised a person‐centred approach that was minimally burdensome, and so often prioritised initiating potentially disease‐modifying interventions instead of overly encouraging individuals to complete screening. The complex psychosocial factors relevant to adults with EOT2D may similarly explain the limited numbers of individuals (32%) responding to and accepting DSN invitations to the high‐risk clinic.

Finally, we were unable to report the distribution of appointments attended in the service across the cohort as the EMR database used for evaluation did not have this level of granularity. Overall, individuals received between 1 and 3 appointments in the high‐risk service, which was generally based on clinical need, but it is also possible that data reflect care received outside of the service, particularly as most individuals were referred to other secondary care clinics and may consequently have received a further appointment by 12‐months duration.

### 4.2 Study results: Interpretation in relation to previous literature

Our novel EOT2D service was associated with benefits to multiple risk factors for adverse long‐term outcomes in people with EOT2D. While it is unclear exactly what caused improvements in cardiometabolic outcomes seen in this study, there was a significant increase in the number of individuals utilising GLP‐1 RAs or tirzepatide (a dual GLP‐1 and glucose‐dependent insulinotropic polypeptide [GIP] receptor agonist), a total of 32% and 20%, respectively, at 12 months. A post‐hoc analysis of the SURPASS programme of trials investigated the use of tirzepatide in adults with EOT2D.^26^ The findings mirrored changes in cardiometabolic outcomes within our service, with a mean HbA1c reduction of 2.5%/28 mmol/mol, body weight reduction of 14 kg and systolic blood pressure reduction of 6 mmHg after 40 weeks of tirzepatide use in SURPASS‐2. While changes in weight were associated with the service, these were relatively modest considering the changes in HbA1c and the increase in the prescription of GLP‐1‐based medications. The reason these changes were not larger is unclear, but it may be related to weight increases in those not taking GLP‐1 RAs (particularly if insulin therapies had been up titrated). Furthermore, a post‐hoc analysis of the SURPASS‐4 study, investigating tirzepatide treatment versus insulin glargine in individuals with type 2 diabetes at high cardiometabolic risk showed that tirzepatide was less effective in achieving weight reduction in individuals with high baseline HbA1c.^27^

In contrast to the increase in GLP‐1‐based medication use observed, there was no difference in the proportion of individuals taking sodium‐glucose co‐transporter 2 inhibitors (SGLT2i) at baseline and 12 months, despite SGLT2i being protective of cardiovascular risk. This is likely to reflect clinician concerns of diabetic ketoacidosis, a rare complication of SGLT2i use which may be more likely in individuals with high HbA1c and potential insulin deficiency. While unclear, other potential reasons include a potential sentiment in this population against initiating new therapies, which may be particularly relevant to second‐line therapies, especially if glucose fell within target range; contraindication in individuals planning pregnancy; and less opportunity to initiate medications with increasing time from baseline due to the sudden loss of service funding. Despite this low uptake, SGLT2i remain important therapies in this population due to high lifetime cardiorenal risk.

A further reason for clinical improvements observed in our service could be the flexible patient‐centred approach, which may have maximised engagement and delivery of care. This is suggested by positive feedback amongst a limited number of individuals responding and acceptable rates of non‐attendance and drop‐out given the complexities faced by young adults with chronic disease. This is also consistent with findings from previous qualitative work that identified an unmet need of access to specialist care.^28^ The study further concluded that this access could be supported by integrated and synchronous health systems that adopt streamlined referrals and interprofessional collaborations, tailored to individuals with EOT2D and are underpinned by person‐centred care and shared decision making. This work reflects the standard inflexible approach to care of adults with EOT2D in the United Kingdom, which is generally delivered in primary care and not targeted to the specific challenges faced by people with EOT2D. Furthermore, access to interventions can be limited in non‐specialist settings, including diabetes education and GLP‐1 RAs as examples. A more holistic person‐centred approach to care, which includes a more intensive focus on cardiometabolic management, as well as addressing barriers to care, is currently being formally tested in the M3 randomised controlled trial.^28^, ^29^

Our service included a focus on women's health and we supported many women with EOT2D, of whom most were likely to be at risk of pregnancy due to not using contraception, as demonstrated by our previous work in a similar cohort.^20^ While we did not have sufficient statistical power to stratify our results across sex, our evaluation strongly suggests that cardiometabolic risk, including body weight, can be improved through specialist person‐centred care in a population including a large proportion of women likely to be planning pregnancy. These improvements could reduce the risk of adverse pregnancy‐related outcomes, although this requires further research. Our recent systematic review highlighted a lack of preconception interventional studies in this patient population,^30^ although narrative synthesis of observational studies suggested that preconception care was associated with increased use of folic acid and vitamin D, and reduced gestational weight gain.^31^ Addressing weight pre‐pregnancy is likely to be important, since higher BMI was associated with multiple suboptimal outcomes in women with EOT2D, affecting pregnancy, birth and neonatal outcomes.^31^ However, there is limited data on optimal use of GLP‐1 agonists and other weight loss strategies pre‐pregnancy in women with EOT2D, and how these should be safely stopped when planning to conceive. Although our service was not primarily designed to increase access to pre‐pregnancy planning, our multidisciplinary clinic addressed multiple risk factors providing an excellent opportunity to optimise future reproductive health and pregnancy outcomes, in addition to supporting long‐term optimal cardiometabolic health in women with diabetes. While our data show that <5 women (out of 34 with a first appointment >12 months prior to the date of analysis) were taking folic acid at 12 months, this is likely because individuals with EOT2D were generally counselled against pregnancy, while HbA1c was very high due to the high risk of associated complications, with the plan for this to be continually rediscussed as HbA1c decreased. Nevertheless, given that approximately half of pregnancies are unplanned and that hyperglycaemia is a recognised risk factor for neural tube defects,^32^ earlier consideration of folic acid initiation may represent an opportunity for service improvement.

It was also noteworthy that high numbers of individuals were referred into other secondary‐care diabetes clinics after attending the high‐risk clinic. This was in part due to an unexpected loss of service funding in September 2025, meaning that individuals having only received one appointment and awaiting further follow‐up (which could have led to eventual discharge to primary care) were instead moved to other non‐bespoke diabetes clinics. It is possible that these individuals were soon discharged from secondary care services although we were unable to capture this in our evaluation. This high number is also likely to reflect the high clinical need of this population, and concerns that clinicians may have had discharging to non‐specialists in primary care given the clinical challenges of working with this population. Despite the large overall reductions in HbA1c, only 55% had HbA1c <8.5%/69 mmol/mol at 12‐months, with even fewer meeting lower thresholds, which may have further justified continual support in more specialist services—particularly as there is growing recognition that a lower HbA1c target (≤6.5%/48 mmol/mol) may be more appropriate for this population.^33^ It is deeply concerning that funding of EOT2D services via the T2Day programme was stopped given the higher lifetime risks in this population and evidence that current care models are not meeting individuals' needs.^14^, ^28^ In practice, this means that individuals within our service are now being seen in non‐bespoke clinics and competing for funding alongside other clinical services within the broader financial constraints of the National Health Service.

Due to the observational nature of this service evaluation, the true impact of our service remains unknown. However, RCTs involving individuals with EOT2D are severely lacking,^34^ with individuals with EOT2D representing <5% of participants in large pharmacological or lifestyle trials.^35^ Therefore, this evaluation provides crucial, albeit non‐interventional, evidence in lieu of RCTs. This evidence is essential as there is a clear need for health systems to immediately improve care and outcomes due to the increasing prevalence of EOT2D and its high burden of associated complications, and evidence generation from RCTs requires a large cost and time. We provide a framework for how secondary care clinics could be implemented in current health systems. Pragmatic service evaluations such as ours provide timely, actionable evidence to inform commissioning decisions and support scalable redesign of EOT2D services within constrained health systems.

Following this evaluation, we recommend that bespoke services for adults with EOT2D are implemented in other centres. From our reflections, we recommend that clinicians and commissioners especially consider the busy lives, competing demands and high burden of mental health in this population. This is likely to mean that important components include the availability of out of hours appointments, flexibility with appointment non‐attendance and ensuring access to psychological and psychiatric care. While our service was initially designed as a ‘one‐stop’ clinic, in practice most individuals were subsequently referred into general secondary care diabetes clinics. Other services looking to implement our approach could perhaps streamline processes by incorporating the key features into existing secondary care services and referring eligible individuals directly, thus bypassing the one‐stop design. Similarly, key aspects of our service may be similarly effective when implemented in primary care, with potential advantages of community integration—including access to social prescribers—and better‐suited geographical locations to improve attendance and engagement, although input from specialist clinicians is likely to be advantageous. A further challenge was that many individuals were against taking medications and wanted a lifestyle‐only approach to management (generally against medical advice given high HbA1c). Routine use of short‐term continuous glucose monitoring may have helped individuals better understand hyperglycaemia and perhaps facilitate lifestyle change and medication initiation in these adults, which could be an addition in future services.

## 5 CONCLUSION

Multidisciplinary clinics for adults with EOT2D at high risk of complications were associated with improvements in cardiometabolic outcomes, and a limited number of patients reported satisfaction with the care received. This work provides a template for other health providers to potentially implement and improve services for adults with EOT2D which are immediately required, given the rising prevalence of EOT2D and its associated complications.

## CONFLICT OF INTEREST STATEMENT

Professor Samuel Seidu is in receipt of speaker honoraria from AstraZeneca, Boehringer Ingelheim, Janssen, Lilly, MSD, Abbott, Novo Nordisk, SB Communications, OmniaMed Communications, Roche, Napp Pharmaceuticals, NB Medical and Amgen; advisory board honoraria from AstraZeneca, Lilly, Boehringer Ingelheim, Janssen, Abbott, MSD, Novo Nordisk, Takeda and Sanofi; educational grants from Boehringer Ingelheim, Lilly, Novo Nordisk and Takeda; and conference registration and subsistence from Boehringer Ingelheim, Janssen, Lilly, Novo Nordisk, Abbott and Takeda. Professor Melanie Davies has acted as a consultant/advisor and speaker for Eli Lilly, Novo Nordisk and Sanofi, as a consultant/advisor for Kailera and has attended advisory boards for AbbVie, Amgen, AstraZeneca, Biomea Fusion, Carmot/Roche, Daewoong Pharmaceutical, Sanofi, Zealand Pharma, Regeneron, GSK, Innovent Bio and EktaH and as a speaker for AstraZeneca, Boehringer Ingelheim and Zuellig Pharma. She has received grants from AstraZeneca, Boehringer Ingelheim and Novo Nordisk. James Ridgeway has received speaker honoraria from Abbott, Eli Lilly, Dexcom and Insulet. He has also received honoraria from Dexcom and Abbott as an advisory board member. Victoria Alabraba is employed by Insulet International; her involvement in this work occurred only during her previous employment at the University Hospitals of Leicester NHS Trust. All other authors have no conflicts of interest to declare.

## Supporting information


**Table S1.** Read codes used to categorise ethnicity groups.
**Table S2.** Days between first visit and baseline, 6‐ and 12‐ month continuous measurements.
**Table S3.** Number and proportion achieving HbA1c thresholds across time‐points.
**Table S4.** Patient feedback—free‐text responses.
