# Supplementary material for: Evaluation of a secondary care multidisciplinary clinic for adults with early‐onset type 2 diabetes at high risk in Leicester, Leicestershire and Rutland
Source: Diabet Med. 2026 Apr 30;43(11):e70347. doi: 10.1111/dme.70347 (PMC13669973; doi:10.1111/dme.70347)
Supplement: Supplementary file 1 [file DME-43-e70347-s001.docx]

Evaluation of a secondary care one-stop clinic for adults with early-onset type 2 diabetes in Leicester, Leicestershire and Rutland

**Supplementary Material**

Contents

[**Supplementary Table S1:** Read codes used to categorise ethnicity groups	2](#_Toc224287570)

[**Supplementary Table S2**: Days between first visit and baseline, 6- and 12- month continuous measurements	3](#_Toc224287571)

[**Supplementary Table S3**: Number and proportion achieving HbA1c thresholds across time-points	4](#_Toc224287572)

[**Supplementary Table S4:** Patient feedback – free-text responses	5](#_Toc224287573)

# **Supplementary Table S1:** Read codes used to categorise ethnicity groups

| **Ethnicity Category** | **Read Code** |
| --- | --- |
| **Black** | (9S3..) Black African |
|  | (XaFwH) Black - ethnic group |
|  | (XaJR6) Caribbean - ethnic category 2001 census |
|  | (XaJR7) African - ethnic category 2001 census |
|  | (XaJR8) Other Black background - ethnic category 2001 census |
|  | (Xactl) Black/African/Carib/Black Brit: African - Eng+Wales 2011 cens |
|  | (Xactn) Black/Afr/Carib/Black Brit: other Black - Eng+Wales 2011 cens |
| **South Asian** | (9S6..) Indian |
|  | (9S7..) Pakistani |
|  | (9S8..) Bangladeshi |
|  | (9SA7.) Indian sub-continent (NMO) |
|  | (XaJR2) Indian or British Indian - ethnic category 2001 census |
|  | (XaJR3) Pakistani or British Pakistani - ethnic category 2001 census |
|  | (XaJR4) Bangladeshi or British Bangladeshi - ethn categ 2001 census |
|  | (XaJRR) Sri Lankan - ethnic category 2001 census |
|  | (Xactg) Asian/Asian Brit: Indian - Eng+Wales ethnic cat 2011 census |
|  | (Xacth) Asian/Asian British:Pakistani - Eng+Wales eth cat 2011 census |
|  | (Xacti) Asian/Asian Brit: Bangladeshi - Eng+Wales eth cat 2011 census |
|  | (Xactk) Asian/Asian Brit: other Asian - Eng+Wales eth cat 2011 census |
| **White** | (XaFwD) White British |
|  | (XaJQv) British or mixed British - ethnic category 2001 census |
|  | (XaJQx) Other White background - ethnic category 2001 census |
|  | (XaQEa) White British - ethnic category 2001 census |
|  | (XactH) White:Eng/Welsh/Scot/NI/Brit - England and Wales 2011 census |
| **Other** | (9SA8.) Other Asian (NMO) |
|  | (XaFwz) Asian - ethnic group |
|  | (XaFx0) Other Asian ethnic group |
|  | (XaJQy) White and Black Caribbean - ethnic category 2001 census |
|  | (XaJQz) White and Black African - ethnic category 2001 census |
|  | (XaJR0) White and Asian - ethnic category 2001 census |
|  | (XaJR5) Other Asian background - ethnic category 2001 census |
|  | (XaJRW) Other Asian or Asian unspecified ethnic category 2001 census |
|  | (XaJRe) Filipino - ethnic category 2001 census |
|  | (XaJSO) Other mixed White - ethnic category 2001 census |
| **Unknown** | (9S...) Ethnic groups (census) |
|  | (XaJQu) Ethnic category - 2001 census |
|  | (XaJRB) Ethnic category not stated - 2001 census |
|  | (No available code) |

# **Supplementary Table S2**: Days between first visit and baseline, 6- and 12- month continuous measurements

| **Measurement** | **Baseline** | **6 months** | **12 months** |
| --- | --- | --- | --- |
| **BMI** | -162 (-274, -51) | 167 (127, 246) | 314 (289, 402) |
| **Weight** | -164 (-279, -54) | 160 (121, 246) | 318 (288, 404) |
| **Systolic blood pressure** | -171 (-280, -79) | 162 (118, 243) | 303 (284, 361) |
| **Diastolic blood pressure** | -165 (-280, -70) | 165 (118, 244) | 303 (284, 361) |
| **HbA1c** | -133 (-262, -54) | 154 (114, 218) | 325 (290, 399) |
| **LDL** | -116 (-246, -41) | 157 (121, 216) | 334 (306, 399) |
| **HDL** | -191 (-280, -128) | 161 (113, 212) | 352 (323, 376) |
| **TG** | -125 (-247, -54) | 158 (125, 224) | 332 (302, 399) |
| **Creatinine** | -174 (-274, -70) | 153 (120, 199) | - |
| **UACR** | -132 (-237, -36) | 154 (126, 197) | 367 (328, 421) |

Values shown represent the median (IQR) days between the first clinic appointment and the closest measured value within the time window (-365 to 7 days for baseline measures, between +91 and +274 days for 6-month measures, between +275 and +457 days for 12-month measures).

**BMI:** Body mass index; **LDL:** Low-Density lipoprotein cholesterol; **HDL:** High-density lipoprotein cholesterol; **TG:** Triglycerides; **UACR:** Urine albumin-to-creatinine ratio

# **Supplementary Table S3**: Number and proportion achieving HbA1c thresholds across time-points

| **HbA1c threshold** | **Baseline* – n (%)** | **6 months – n (%)** | **P-value for difference at 6 months** | **12 months – n (%)** | **P-value for difference at 12 months** |
| --- | --- | --- | --- | --- | --- |
| **<6.5% / 48 mmol/mol** | <5 | 19 (16.8) | 0.001 | 20 (28.2) | <0.001 |
| **<7.5% / 58 mmol/mol** | 10 (6.9) | 40 (35.4) | <0.001 | 27 (38.0) | <0.001 |
| **<8.5% / 69 mmol/mol** | 18 (12.4) | 56 (49.6) | <0.001 | 39 (54.9) | <0.001 |

Values shown represent the number of individuals achieving HbA1c thresholds, and the proportion (%) of individuals meeting the threshold of the total with HbA1c recorded at that timepoint: 145 individuals had HbA1c recorded at baseline, 113 at 6 months, and 71 at 12 months.

*Despite the criteria for the clinic being HbA1c >9/10%, it was possible for the HbA1c to be lower at baseline due to delays between referral acceptance and the first appointment in the clinic.

# **Supplementary Table S4:** Patient feedback – free-text responses

**Question 4: Is there any way we could improve the service? Is there anything else you would like to say about the service?**

Improve it no, works very well. Service so far is top notch. Without this help, i don't think i would have been able to do what is done so far. A massive THANK YOU!

It would be great to be able to speak with the Drs at the Diabetes clinic when we have concerns instead of talking to our GP.

More availability if possible

Nice

No comment

No your service and staff are wonderful

No, im extremely happy. My type 2 diabetes is under control and meds now reduced by my gp

The entire team have been amazing! Blood forms get a little confusing sometimes but nothing to complain about as I always get the support needed to manage them. Thank you for all your hard work and quality care. Genuinely, thank you.

The full information and support hadn't been provided when I initially started ( a few months after my diagnosis).

Note: one response was removed due to containing potentially identifiable information.
